# Human behaviour can trigger large carnivore attacks in developed countries

**DOI:** 10.1038/srep20552

**Published:** 2016-02-03

**Authors:** Vincenzo Penteriani, María del Mar Delgado, Francesco Pinchera, Javier Naves, Alberto Fernández-Gil, Ilpo Kojola, Sauli Härkönen, Harri Norberg, Jens Frank, José María Fedriani, Veronica Sahlén, Ole-Gunnar Støen, Jon E. Swenson, Petter Wabakken, Mario Pellegrini, Stephen Herrero, José Vicente López-Bao

**Affiliations:** 1 Department of Conservation Biology, Estación Biológica de Doñana, C.S.I.C., c/Américo Vespucio s/n, 41092 Seville, Spain; 2 Research Unit of Biodiversity (UMIB, UO-CSIC-PA), Oviedo University-Campus Mieres, 33600 Mieres, Spain; 3 Metapopulation Research Centre, University of Helsinki, FI-00014 Helsinki, Finland; 4 C.I.S.D.A.M., Via S. Liberata 1, Rosello (CH) I-66040, Italy; 5 Natural Resources Institute Finland, P.O. Box 16, FI-96301 Rovaniemi, Finland; 6 Finnish Wildlife Agency, Sompiontie 1, FI-00730 Helsinki, Finland; 7 Grimsö Wildlife Research Station, Department of Ecology, Swedish University of Agricultural Sciences, 73091 Riddarhyttan, Sweden; 8 Centre for Applied Ecology “Prof. Baeta Neves”, Institute Superior of Agronomy, University of Lisbon, Tapada da Ajuda, 1349-017 Lisboa, Portugal; 9 Department of Ecology and Natural Resource Management, Norwegian University of Life Sciences, Postbox 5003, NO-1432 Ås, Norway; 10 The Norwegian Environment Agency, P.O. Box 5672 Sluppen, N-7485 Trondheim, Norway; 11 Faculty of Applied Ecology and Agricultural Sciences, Hedmark University College, Evenstad, NO-2480, Koppang, Norway; 12 Faculty of Environmental Design, University of Calgary, Calgary, Alberta, Canada T2T 2Y2

## Abstract

The media and scientific literature are increasingly reporting an escalation of large carnivore attacks on humans in North America and Europe. Although rare compared to human fatalities by other wildlife, the media often overplay large carnivore attacks on humans, causing increased fear and negative attitudes towards coexisting with and conserving these species. Although large carnivore populations are generally increasing in developed countries, increased numbers are not solely responsible for the observed rise in the number of attacks by large carnivores. Here we show that an increasing number of people are involved in outdoor activities and, when doing so, some people engage in risk-enhancing behaviour that can increase the probability of a risky encounter and a potential attack. About half of the well-documented reported attacks have involved risk-enhancing human behaviours, the most common of which is leaving children unattended. Our study provides unique insight into the causes, and as a result the prevention, of large carnivore attacks on people. Prevention and information that can encourage appropriate human behaviour when sharing the landscape with large carnivores are of paramount importance to reduce both potentially fatal human-carnivore encounters and their consequences to large carnivores.

During the last few decades, large carnivore attacks on humans in developed countries have increased over time^1^,^2^,^3^,^4^,^5^,^6^,^7^,^8^ (Fig. 1). This is expected to increase people’s apprehension and reduce their willingness to share the landscape with large carnivores. Unfortunately, such rare events are usually overplayed by the media. Indeed, media coverage of such attacks generally includes sensational texts and dreadful pictures (Extended Data 1), appealing more to the public’s emotions than their logic. Denominator neglect^9^ is a well-studied phenomenon leading humans to overestimate the risk of rare events that evoke strong emotions. Overestimating the risk of large carnivore attacks on humans irrationally enhances human fear and triggers a vicious cycle that may affect the increasingly positive conservation status of many of these contentious species^10^,^11^,^12^. With an increasing number of large carnivore attacks on humans there is, now more than ever, a need for objective and accurate information regarding not only the long-term trend and underlying mechanisms of large carnivore attacks on humans, but also potentially risky situations and risk-enhancing human behaviours^8^. Surprisingly, the few available studies focus on attacks by single carnivore species and thus they do not provide a comprehensive perspective concerning the pervasiveness and socio-ecological correlates of this phenomenon in developed countries.

Our main hypothesis is that lack of knowledge of people about how to avoid risky encounters with large carnivores engenders risk-enhancing behaviours, which can determine an increase in the number of attacks if more humans are sharing landscape with large carnivores. Three main predictions arise from this hypothesis: (1) an increased number of people are engaging in outdoor leisure activities in areas inhabited by large carnivores; (2) many people are not prepared to safely enjoy outdoor activities or they behave inappropriately in the countryside; and (3) large carnivore attacks are influenced by the interaction between several human- and animal-related factors.

Thus, we first explored whether the long-term patterns in the number of attacks have been similar among different large carnivore species, and how they varied throughout the year. Then, we evaluated whether there might have been a general long-term change in the attack patterns by assessing whether victim ages and the frequency of attacks on parties *vs*. lone humans have changed in a congruent manner for the different species. Finally, we assessed the possible relationships between temporal trends of attacks on humans and outdoor activities, as well as the role that risk-enhancing human behaviour can have played in the observed increase in large carnivore attacks.

We analysed the circumstances of ca. 700 large carnivore attacks on people from 1955 in detail (when more reliable data became available) until the present for six species responsible for most of the large carnivore attacks recorded in North America and Europe: brown/grizzly bear *Ursus arctos*, black bear *Ursus americanus*, cougar *Puma concolor*, wolf *Canis lupus* and coyote *Canis latrans* in North America, brown bear in Europe, and polar bear *Ursus maritimus* circumpolar, i.e. Europe, Russia and North America. We also collected statistics concerning outdoor activities during the same period (see Methods for more details).

The number of large carnivore attacks on people has increased significantly over time, with contrasting trends across species (Fig. 1; Extended Data Table 1). In North America, coyotes (31.0% of the total number of attacks) and cougars (25.7%) were responsible for the majority of attacks, followed by brown bears (13.2%), black bears (12.2%) and wolves (6.7%). A similar increase over time was observed for brown bears (9.3%) in Europe and circumpolar for polar bears (1.9%; Fig. 1). Moreover: (a) the age of victims has also increased significantly over time showing different patterns across species (Extended Data Table 2 and Extended Data Fig. 1); and (b) the propensity to attack lone humans or parties depends on the large carnivore species, and only a slight but non-significant increasing trend of attacks on parties has been observed (Extended Data Table 3 and Extended Data Fig. 2A,B).

The patterns of attacks reported here may also reflect an increasing number of bold individuals in large carnivore populations, as this trait is often correlated with aggressiveness^13^,^14^, and this might lead to more aggressive responses when large carnivores encounter humans. We hypothesise that intense and prolonged human-caused mortality imposes selection pressures on target populations (selective removal of certain phenotypes) and might lead to rapid evolutionary changes^15^. Natural selection maintains a mix of behavioural phenotypes in populations^16^, the shy-bold behavioural continuum^17^; bold individuals thrive on risk and novelty, whereas shy individuals shrink from the same situations^18^. Persecution, however, is expected to result in the disproportionate removal of bold individuals, as they are less cautious^19^, and thus more likely to be killed. As a consequence, shy individuals might have been overrepresented in remnant large carnivore populations in the past^17^,^18^,^20^,^21^,^22^. Additionally, individuals may become more vigilant and actively avoid contact with humans during times of intense persecution^23^. Although the history of large carnivore persecution and conservation differ across regions^9^, the contemporary conservation paradigm emerged during the 1960s–1970s^24^, when most bounty systems were banned^25^ and large carnivores were reclassified from vermins or bountied predators to game or protected species. Since then, although large carnivores have continued to be hunted or managed (Extended Data Fig. 3), most populations have generally increased during the past four decades^9^,^11^,^12^. Increasing population trends in conjunction with relaxed artificial selection may potentially engender higher variation in behavioural temperaments^26^, which is likely to alter individual responses to human encounters^22^. This significant increase of large carnivore populations in both North America and Europe, and their consequent range expansion, also may contribute to explain the observed increase in the attacks on humans.

However, similar to the increasing trend in attacks, the number of people engaging in outdoor leisure activities also has risen over time, a phenomenon that is significantly correlated with the observed trend in the number of attacks (Fig. 1; Extended Data Table 4, Extended Data Fig. 4A–C). Seasonally, most of the attacks occurred between late spring and early autumn (Fig. 2), when most people pursue outdoor activities^7^,^8^; in addition, because bears hibernate, they are unlikely to attack people in winter. Such an increase in recreational activities in areas inhabited by large carnivores implicitly increases the probability of a risky encounter and, therefore, a potential attack. However, even with more people visiting those areas, attacks are still extremely rare (Fig. 1): although some people may only focus on the total number of attacks, we have to bear in mind the long time period during which these attacks occurred.

Remarkably, risk-enhancing human behaviour has been involved in at least half of the well-documented attacks (47.6%; Fig. 3). From highest to lowest, the five most common human behaviours occurring at the time of an attack were (a) parents leaving children unattended, (b) walking an unleashed dog, (c) searching for a wounded large carnivore during hunting, (d) engaging in outdoor activities at twilight/night and (e) approaching a female with young. These are clearly risk-enhancing behaviours when sharing the landscape with large carnivores. For example, the most frequently recorded human behaviour was children left unattended (47.3%), which were most often attacked by cougars (50.8% of the attacks), coyotes (27.9%) and black bears (13.2%). Risk-enhancing human behaviour is not the sole reason behind large carnivore attacks on humans. The causes of the other half of the attacks do not seem to be related to risk-enhancing human behaviour, for example, accidentally walking close to a mother with young or to a carcass with a bear nearby or an encounter with a food-conditioned individual (which is an indirect result of a risk-enhancing human behaviour^8^).

Thousands of interactions occur between people and large carnivores with no human injuries or fatalities. Even if attacks have increased over time, they remain extremely rare events (e.g. a cross-continental average of 24.1 attacks and 3.9 fatalities per year during the last decade, all species pooled; Fig. 1). Other wildlife (bees and mosquitos, spiders, snails, snakes and ungulates) and domestic dogs are far more responsible for human fatalities^1^,^27^. But humans are not the only victims. When attacks occur, large carnivores are frequently killed and negative attitudes towards large carnivores harden^6^. Lethal removal of ‘problematic’ individuals is effective in solving the local problem caused by a given individual^28^, but generally this happens after an aggressive behaviour, human injury or death has occurred. Consequently, both humans and carnivores suffer from these incidents.

After decades of minimal interaction between humans and large carnivores in many regions of developed countries, many people involved in outdoor activities may lack knowledge about how to avoid risky encounters with large carnivores and what to do when such encounters occur. From an early age most of us learn social norms, rules and how to decrease risks in urban environmental settings, but much less effort is expended to teach us how to safely enjoy outdoor activities or to behave appropriately in the countryside. However, it is up to us to reduce the likelihood of an attack. The increasing human presence in areas inhabited by large carnivores, together with their population recoveries^9^,^11^,^12^, requires an improvement in information, education and prevention guidelines, and their enforcement, which are of paramount importance to reduce both the risks to humans and the killing of carnivores^1^,^4^,^7^,^28^,^29^. Educating people that share landscape with large carnivores can represent a crucial factor to help reducing the number of attacks and also the negative attitudes towards large carnivore conservation, especially because of the difficulty to envisage risk estimates. Indeed, scenarios of attacks are extremely different and may depend on many different factors, such as human population and carnivore densities, time of the day, human activities, personality and condition of the large carnivore, party size or even subtle details, like the presence of an unleashed dog at the moment of the attack and/or the landscape features of the area where an attack has happened. As conflicts between humans and large carnivores continue to increase, accurate information becomes crucial to informed human–wildlife conflict management. Communicating about large carnivore-inflicted human injuries and fatalities in a statistical manner contributes to better understanding of common patterns in large carnivore attacks, further reduces chances of injury or death and promotes public appreciation of these species. An important strategy to reduce attacks on humans is to inform people how to avoid and manage aggressive encounters. But nowadays, educational and interpretive efforts aimed at decreasing the risk of large carnivore attacks should not focus exclusively on people living in rural and wilderness areas. Indeed, many people living in cities should also be included within the category of groups at risk because of the increasing number of them enjoying outdoor activities in areas inhabited by large carnivores and the expanding population of carnivores (mainly coyotes) in suburban areas.

Although large carnivore attacks on humans are influenced by the interaction between multiple human- and animal-related factors, adapting our own behaviours when coexisting with large carnivores has the potential to reduce the number of attacks to about half of today’s level. The examples provided by the numerous cases of children injured/killed while left unattended by their parents, attacks on people jogging/walking alone at twilight and during hunting, should make us reflect on our responsibilities, the possibility of decreasing the number of these tragic events and changing the observed trends. Understanding the circumstances associated with large carnivore attacks should help us to reduce them and thereby minimize the role that fear and supposition may play in large carnivore management and conservation.

## Methods

### Collection of records of large carnivore attacks on humans

Records of large carnivore attacks (i.e. attacks resulting in physical injury or death) on humans for the brown bear, black bear, cougar, wolf and coyote were collected for North America (the United States and Canada). In addition, with the aim to broaden our research and obtain a general picture of large carnivore attacks on humans in developed countries, we complemented the North American dataset with information on brown bear attacks in three European countries (Sweden, Finland and Spain) as well as data on attacks by polar bears in Europe (Svalbard; Norway), Russia, the United States, and Canada. Our time period spanned from 1955 to 2014 and our search resulted in a total of 697 attacks of large carnivores on people.

We consider that we both recorded the majority of such events occurring during the last six decades in these developed countries and avoided bias due to possible changes in reporting probability given (*i*) the large number of experienced people involved in the work (some of them had their own database on attacks, which started at the beginning of the 1900s), (*ii*) the multiple sources of information used to collect recorded attacks and (*iii*) the sensational nature and media impacts of attacks that end with injuries or the death of the victim since the beginning of the past century. Records of attacks were collected from unpublished reports and PhD/MS theses, webpages (last accessed in November 2014, but currently available at the specific addresses listed by species below), books and scientific articles, as well as personal datasets from some of the co-authors. In addition, to complete the data obtained from the above-cited sources, we also collected dozens of news reports from online newspapers. To do this, for each species and area, we searched on an annual basis for news articles on Google using the combination of the following terms: “species name” + “attack” and “species name” + “attack” + “human”. Because of the use of multiple sources, several attacks recurred repeatedly during the search, but we used information such as date, locality and sex/age of the victims to prevent duplicate records in the dataset. However, the lack of some records would still not result in a bias in the general patterns we observed in the present work, because: (*i*) we followed the same procedure for each species and, thus, we collected at least an equally biased sample of attacks per species and (*ii*) patterns of attacks on humans over time are less sensitive to unequally biased samples of attacks than quantitative comparisons of the frequency of attacks across species (which is not the aim of the present work).

When possible, we recorded the following information for each attack: (1) species; (2) year; (3) month; (4) country; (5) time of the attack during the day (which we classified into three categories: twilight, day, night); (6) activity of the victim (15 categories: hunting, fishing, field work, camping, hiking, jogging, skiing, biking, horse riding, fruit/mushroom picking, photography, walking, dog walking, activity near the house/in the backyard, playing); (7) size of party being attacked (simplified into three categories: victim alone, child – from 0 to 16 years old– in a party of adults, adult – >16 years old – in a party of adults); (8) end of the attack, i.e. attack resulting in human injuries or death; and (9) scenario when the attack occurred, i.e. the factor that could have triggered the attack. We were able to delineate eight categories: female with young, aggressive reaction after a sudden encounter (i.e. a person surprises the large carnivore at close range), food defence (e.g. a bear close to a carcass), food conditioning (i.e. encounter with a large carnivore that consumes human-derived foods, consequently associating people with easily accessible, attractive foods, and which has lost much of its avoidance mechanisms towards humans), predatory (i.e. when the large carnivore exploited a human as prey), wounded animal (i.e. during hunting), feeding large carnivores, and presence of one or more dogs. Unleashed dogs can exacerbate the probability of a large carnivore attack, because a dog that runs away from a large carnivore towards the owner can trigger a dangerous situation when the carnivore chases it^30^. When dogs were involved, large carnivores usually focused their attention on the dog rather than on the person. However, in some instances the human was attacked as a consequence of its proximity to the dog or because of its reaction towards the large carnivore.

Below, we describe the sources used to collect data on large carnivore attacks on people for each species since 1955:

1. *North American brown and black bears*. We recorded a total of 92 and 85 attacks, respectively. Information was compiled from^28^, Wikipedia List of fatal bear attacks in North America ( http://en.wikipedia.org/wiki/List_of_fatal_bear_attacks_in_North_America), Fatal Bear Attack Statistics for the USA & Canada ( http://www.blackbearheaven.com/bear-attack-statistics.htm) and online newspapers. Additionally, we also extracted information for the black bear from^8^ and California Black Bear Public Safety Incidents, California Department of Fish and Wildlife ( https://www.wildlife.ca.gov/News/Bear/Bear-Incidents).
2. *Cougar*. We recorded a total of 179 attacks. Data on attacks were collected from^1^,^31^, the List of Mountain Lion Attacks ( http://www.cougarinfo.org/attacks.htm), Mountain Lion Attacks from 1991 to 2000 ( http://www.cougarinfo.org/attacks2.htm), Mountain Lion Attacks from 2001 to 2010 ( http://www.cougarinfo.org/attacks3.htm), Mountain Lion Attacks from 2011 to Now ( http://www.cougarinfo.org/attacks4.htm) and online newspapers.
3. *Wolf*. We recorded a total of 47 attacks. Data on attacks were collected from^32^,^33^, the Wikipedia List of wolf attacks in North America ( http://en.wikipedia.org/wiki/List_of_wolf_attacks_in_North_America), Wikipedia List of wolf attacks ( http://en.wikipedia.org/wiki/List_of_wolf_attacks), Wolf Attacks on Humans ( http://www.aws.vcn.com/wolf_attacks_on_humans.html) and online newspapers. We did not include the wolf in Europe, because predatory attacks on people have been extremely rare during the last six decades, with the last recorded predatory attack occurring in 1974 in Spain^32^.
4. *Coyote*. We recorded a total of 216 attacks. Data on attacks were collected from^4^,^34^,^35^,^36^, the Wikipedia Coyote attacks on humans ( http://en.wikipedia.org/wiki/Coyote_attacks_on_humans), Coyote Attacks on Children ( http://www.varmintal.com/attac.htm), Coyote Attacks: An Increasing Suburban Problem ( http://escholarship.org/uc/item/8qg662fb), Coyote Attacks On People in the U.S. and Canada ( http://tchester.org/sgm/lists/coyote_attacks.html) and online newspapers.
5. *European brown bear*. We recorded a total of 65 attacks. Information from Spain was available from the unpublished personal database of J.N. and A.F.G., whereas Fennoscandian records were obtained from^37^,^38^ and unpublished data from I.K., H.N. and J.F.
6. *Polar bear*. We recorded a total of 13 attacks. Information was recorded from Wikipedia List of fatal bear attacks in North America ( http://en.wikipedia.org/wiki/List_of_fatal_bear_attacks_in_North_America) and online newspapers (both North American and European –some attacks have been recently recorded in the Norwegian Svalbard archipelago–).

### Collection of records on outdoor human activities

Data on outdoor activities was only available for the US and Sweden. We collected the following information: (1) annual recreation visitation in American Protected Areas published by the National Park Service Visitor Use Statistics (IRMA data system), National Park Service, U.S. Department of the Interior, Natural Resource Stewardship and Science ( https://irma.nps.gov/Stats/Reports/National). To reduce bias in our analyses, we only used information from the National Parks located in the 30 states where at least one large carnivore attack occurred since 1955 (Alaska, Arizona, California, Colorado, Florida, Georgia, Idaho, Illinois, Kansas, Kentucky, Massachusetts, Michigan, Minnesota, Montana, New Hampshire, New Jersey, New Mexico, New York, North Carolina, North Dakota, Ohio, Oklahoma, Pennsylvania, Tennessee, Texas, Utah, Vermont, Virginia, Washington, Wyoming); (2) statistics on number of people doing outdoor activities in the US, which were obtained from^39^, the U.S. Department of the Interior, U.S. Fish and Wildlife Service, and U.S. Department of Commerce, U.S. Census Bureau^40^,^41^,^42^,^43^,^44^; (3) American trends in the sporting goods market related to outdoor activities associated with attacks (cross-country training shoes, jogging and running shoes, camping, optics, snow skiing, bicycles and related supplies), which were collected from the U.S. Census Bureau, Statistical Abstract of the United States^45^,^46^; and (4) Statistics Sweden’s time series tables concerning outdoor activities, which derive from ULF surveys (Living Conditions Surveys) from 1975 onwards ( http://www.scb.se/sv_/Hitta-statistik/Statistik-efter-amne/Levnadsforhallanden/Levnadsforhallanden/Undersokningarna-av-levnadsforhallanden-ULFSILC/12202/12209/#). Information on outdoor human activities was only used to support the highlighted trends and patterns of large carnivore attacks; thus, its sole function is to be supportive to the main text and it was not used in our analyses.

### Collection of records on large carnivore harvest

We used data from brown bear, black bear, cougar and wolf harvests in certain US and Canadian states as examples of trends and numbers in large carnivore harvest over time. First, brown bear harvesting records for Alaska and British Columbia were obtained from the Alaska Department of Fish and Game^47^,^48^ and M. Wolowicz unpublished data (Big Game Harvest Statistics 1976–2012, British Columbia), respectively. Second, data on black bear harvesting statistics in Alaska was obtained from the Alaska Department of Fish and Game^49^,^50^,^51^. Third, cougar harvesting records in Colorado, Alberta and British Columbia were obtained from^52^,^53^,^54^, J. Apker unpublished data (Colorado Division of Wildlife) and M. Wolowicz unpublished data (Big Game Harvest Statistics 1976–2012, British Columbia). Finally, wolf harvesting statistics were extracted from the Alaska Department of Fish and Game^55^,^56^,^57^,^58^. Again, as we did for the information on outdoor human activities, the records on large carnivore harvest were only used to support the highlighted trends and patterns of large carnivore attacks; thus, their sole function is to be supportive to the main text and they were not used in our analyses.

### Data analysis

Considering the total dataset on large carnivore attacks since 1955, we first assessed whether the number of attacks varied over time, on a yearly basis, and among species by fitting a Generalized Linear Model (GLM) with the number of attacks against year and species (Extended Data Table 1). We also included the interaction term between year and species to account for the fact that the number of attacks may vary over time heterogeneously across species. Because our data were overdispersed, we fitted the GLM using a Negative Binomial distribution instead of a Poisson distribution. Next, to assess a potential change in the behavioural temperament of large carnivores over time, we tested whether the log-transformed age of the victim and party size (three levels) varied over time and among species by fitting a linear model with a Gaussian distribution and a GLM with a multinomial distribution (three levels), respectively (Extended Data Tables 2 and 3). Party size was classified into three categories, which allows differentiating between attacks on lone individuals and groups, as well as if the victim in a group was a young person: i) the victim was alone; ii) the victim was a young person (<16 years old) in a group of adults (2 or more people); and iii) the victim was an adult (>16 years old) in a group of adults (2 or more people). We also considered the interaction term in these models to account for the fact that the surrogates of the changes in the temperament of large carnivores used may vary over time differently across species. Finally, we analysed a subset of the dataset considering only those attacks occurring in the US and, together with information on human influx in natural areas, we tested if the number of attacks was related to the number of people involved in outdoor activities by building a GLM with a Gamma distribution, considering year, the number of visitors and their interaction term as factors in the model (Extended Data Table 4).

For each analysis, we used an information theoretic framework to rank a set of competing models based on AIC (Akaike’s Information Criterion [AIC]^59^). We used a stepwise selection procedure to create a candidate set of a priori competing models starting from the simplest null model (intercept only model) to the full model (Extended Data Tables 1, 2, 3 and 4). To select the best candidate model, we used AIC value corrected for small sample sizes (AICc) and Weighted AIC, which indicates the probability that the model selected is the best among the candidates^59^. Models within ΔAIC <2 were considered to have substantial empirical support^59^. All statistical analyses were performed using R 3.0.2 statistical software^60^. GLMs were run with the “lme4”^61^ and “nlme”^62^ package.

## Additional Information

**How to cite this article**: Penteriani, V. *et al.* Human behaviour can trigger large carnivore attacks in developed countries. *Sci. Rep.* **6**, 20552; doi: 10.1038/srep20552 (2016).

## Supplementary Material

Supplementary Information

## Figures and Tables

**Figure 1:**
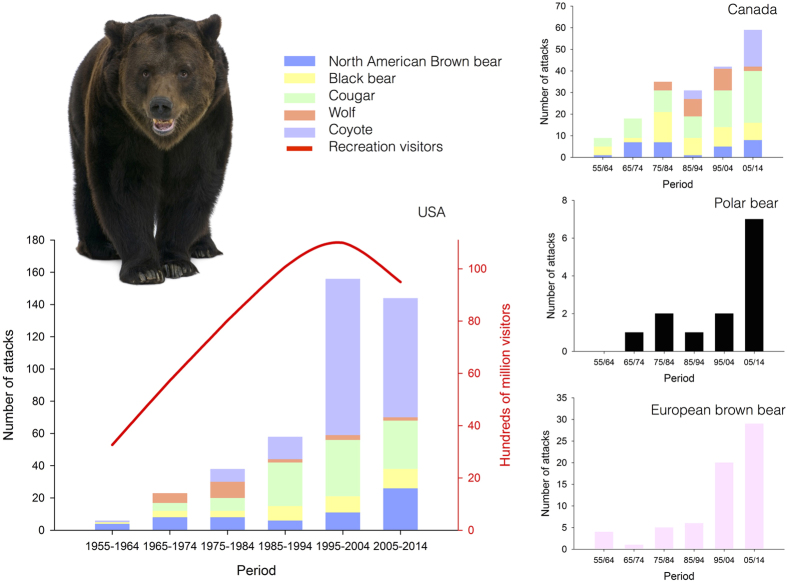
Temporal trends in large carnivore attacks on humans in developed countries. The number of attacks on humans by large carnivores has increased significantly (Extended Data Table 1) during the last few decades for almost all large carnivores. The left panel shows the relationship between the number of large carnivore attacks in the US and the number of visitors (hundreds of millions, red line) in American protected areas since 1955, which has increased significantly over time (Extended Data Table 4). The right panels show (from top to bottom) the temporal trends in large carnivore attacks in Canada, as well as the trends of polar bear (Europe, Russia, the United States and Canada) and European brown bear (Sweden, Finland and Spain) attacks. It is worth noting that: (i) conflicts with polar bears have been increasing in the last decade. Causal factors include a growing human population and more tourists visiting polar bear areas, increased oil and gas development along the Arctic coastline, and decreasing ice volume and seasonal extent due to climate change^63^. Indeed, human-polar bear encounters are expected to increase as the sea ice continues to melt and hungry bears are driven ashore ( http://www.polarbearsinternational.org/about-polar-bears/essentials/attacks-and-encounters; http://www.theguardian.com/world/2013/nov/04/polar-bear-attacks-scientists-warn-warming-arctic); (ii) the remarkable increase in coyote attacks may be related to both the recent substantial expansion of the coyote range in eastern North America^64^ and increased conflicts in suburban residential areas. In these areas, coyotes can relax human avoidance mechanisms as a result of relying on anthropogenic food resources and even intentional feeding by residents^4^; and (iii) wolves were the only species to show a decreasing trend in the number of attacks, declining from 10 attacks during the decade 1975–1984 to only two or three attacks per decade starting in 1985. (The brown bear picture has been downloaded from 123RF ROYALTY FREE STOCK PHOTOS ( http://www.123rf.com), Image ID 7250879, Eric Isselee).

**Figure 2:**
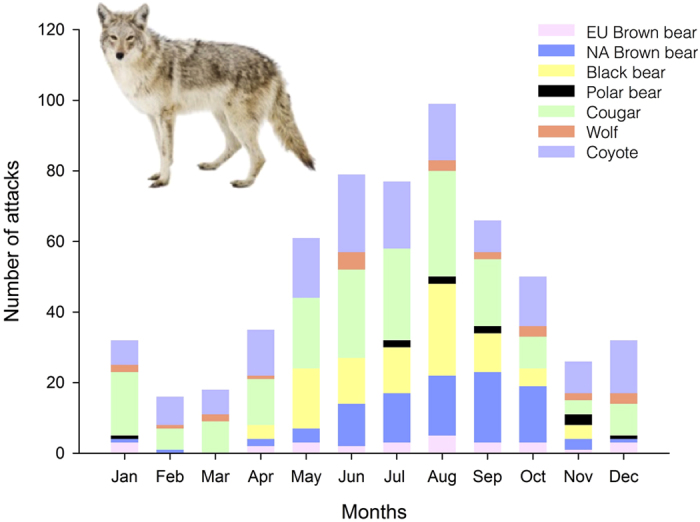
Temporal trends in large carnivore attacks on humans in developed countries: monthly patterns. Most large carnivore attacks occurred from late spring to early autumn, when most people usually engage in outdoor activities. (The coyote picture has been downloaded from 123RF ROYALTY FREE STOCK PHOTOS ( http://www.123rf.com), Image ID 14988151, James Mattil).

**Figure 3:**
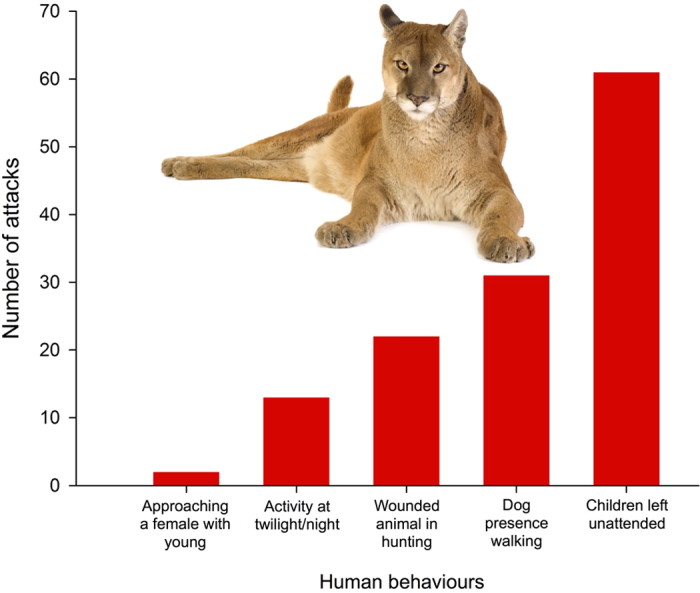
The number of attacks is modulated by human behaviour. Around half of the attacks were associated with risk-enhancing human behaviours. Out of 271 well-documented attacks, 47.6% were associated with certain human behaviours that may have contributed to the probability of suffering an attack. Within the principal category (children left unattended by their parents), the main species responsible for 91.9% of these attacks were cougars (50.8%), coyotes (27.9%) and black bears (13.2%). (The cougar picture has been downloaded from 123RF ROYALTY FREE STOCK PHOTOS ( http://www.123rf.com), Image ID 2597979, Eric Isselee).
