# Corrigendum: Human behaviour can trigger large carnivore attacks in developed countries

**DOI:** 10.1038/srep45250

**Published:** 2017-03-28

**Authors:** Vincenzo Penteriani, María del Mar Delgado, Francesco Pinchera, Javier Naves, Alberto Fernández-Gil, Ilpo Kojola, Sauli Härkönen, Harri Norberg, Jens Frank, José María Fedriani, Veronica Sahlén, Ole-Gunnar Støen, Jon E. Swenson, Petter Wabakken, Mario Pellegrini, Stephen Herrero, José Vicente López-Bao

Scientific Reports 6: Article number: 2055210.1038/srep20552; published online: 02 03 2016; updated: 03 28 2017

This corrigendum aims to correct errors detected in the outputs of the Extended Data Tables from our article Scientific Reports 6, 20552 (2016). A mistake occurred due to a failure in the conversion of the variable “species” from integer to a categorical (factor) variable. We have updated the tables with ‘species’ as a factor (Extended Data Tables below). It is important to state that the essence of our results and conclusions do not change from those presented in the article.

Additionally, we took opportunity of this corrigendum to explore the variation in the group composition (party size) of humans that suffered large carnivore attack over time and across species. We simplified our response categorical variable ‘group composition’ into two categories, i.e., ‘victim alone’ and ‘victim in a party’ (binary coded variable). We conducted this analysis using a two-level categorical approach (binomial family in R) instead of using the previous multinomial approach. This change does not affect the results or conclusions of our analysis, as reported in the article, but is a simplification.

The correct Extended Data Tables 1, 2, 3 and 4 appear below as Table 1, 2, 3 and 4 respectively.

## Figures and Tables

**Table 1:** Variation in the number of large carnivore attacks on humans over time and among species.

COMPETING MODELS		*β*	SE	*p*	AICc	ΔAICc	Weighted AICc
**1a**							
Year + Species + Year: Species					850.5		0.52
	*Intercept*	−0.333	0.453	0.462			
	*Year*	0.024	0.010	0.016			
	*Grizzly*	0.737	0.527	0.162			
	*Black bear*	0.472	0.539	0.381			
	*Cougar*	0.499	0.517	0.335			
	*Wolf*	0.514	0.639	0.421			
	*Coyote*	0.115	0.599	0.848			
	*Polar bear*	−0.398	1.093	0.716			
	*Year: Grizzly*	−0.009	0.012	0.458			
	*Year: Black bear*	−0.008	0.012	0.530			
	*Year: Cougar*	0.006	0.011	0.609			
	*Year: Wolf*	−0.016	0.016	0.326			
	*Year: Coyote*	0.023	0.013	0.071			
	*Year: Polar bear*	0.002	0.023	0.930			
Year + Species					850.6	0.2	0.48
Species					908.1	57.6	0.00
Year					933.5	83.0	0.00
Null model					996.3	145.8	0.00
**1b**							
Year + Species + Year: Species					508.6		0.88
	*Intercept*	0.411	0.284	0.148			
	*Year*	0.015	0.007	0.035			
	*Cougar*	−0.264	0.391	0.500			
	*Coyote*	−0.706	0.524	0.178			
	*Year: Cougar*	0.015	0.009	0.104			
	*Year: Coyote*	0.034	0.012	0.004			
Year + Species					512.6	4.0	0.12
Year					529.4	20.8	0.00
Species					549.7	41.1	0.00
Null model					575.3	66.7	0.00

(1a) Comparison of the five competing models built to study variation in the number of large carnivore attacks on humans over time and among species (n = 231). Summary of fitted parameters is shown for the most parsimonious candidate model (the selected model was the one with the lowest AICc score). Competitive models are ranked from the lowest AICc value (best model) to the highest one. (1b) We present the same analysis, but removed those species showing some patterns in the residuals of 1a. It is worth mentioning that in both cases we obtained the same results. European brown bear is included in the intercept. Negative binomial distribution error was selected over a Poisson distribution error, considering the output of the function odTest from the “pscl” package in R, which compares the log-likelihood ratios of a Negative Binomial regression to the restriction of a Poisson regression (critical value of test statistic at the alpha = 0.05 level: 2.7055; Chi–Square Test Statistic = 10.661, P < 0.001)

**Table 2:** Variation of the age of victims in large carnivore attacks on humans in relation to time and species.

COMPETING MODELS		*β*	SE	*p*	AICc	ΔAICc	Weighted AICc
Year + Species					487.6		0.93
	*Intercept*	−28.016	9.767	0.005			
	*Year*	0.016	0.005	0.001			
	*Grizzly*	−0.543	0.223	0.016			
	*Black bear*	−1.174	0.193	6.65e−09			
	*Cougar*	−1.440	0.306	5.13e−06			
	*Wolf*	−2.021	0.202	<2e-16			
Year + Species + Year: Species					493.2	5.6	0.06
Species					496.1	8.5	0.01
Year					572.9	85.3	0.0
Null model					573.3	85.7	0.0

Comparison of the five competing models built to study the variation in the age of victims over time and across species (n = 188). A summary of fitted parameters is shown for the most parsimonious candidate model. Competitive models are ranked from the lowest AICc value (best model) to the highest one. European brown bear is included in the intercept. Response variable: Log (age of victims)–normal distribution error. Adjusted R-squared = 0.3842.

**Table 3:** Variation of the group composition (party size) targeted in a large carnivore attack on humans over time and across species.

COMPETING MODELS		*β*	SE	*p*	AICc	ΔAICc	Weighted AICc
Species					508.81		0.49
	*Intercept*	−1.335	0.503	0.008			
	*Grizzly*	1.558	0.556	0.005			
	*Black bear*	1.079	0.571	0.059			
	*Cougar*	1.259	0.532	0.018			
	*Wolf*	1.671	0.651	0.010			
	*Coyote*	0.566	0.574	0.324			
	*Polar bear*	1.740	1.042	0.095			
Year + Species					508.91	0.4	0.41
Null model					512.71	4.2	0.06
Year					514.51	6.0	0.02
Year + Species + Year: Species					515.91	7.4	0.01

Comparison of the five competing models built to study the variation of the group composition targeted in an attack over time and across species (n = 371). A summary of fitted parameters is shown for the most parsimonious candidate model. Competitive models are ranked from the lowest AICc value (best model) to the highest one. Group composition was classified into two categories: (1) victim alone and (2) victim in a party. European brown bear is included in the intercept. Response variable: Group composition (2 levels: victim alone and victim in a party)–binomial distribution error. Deviance = 0.032.

**Table 4:** Relationship between the yearly number of large carnivore attacks and the number of recreation visitors in national parks in the US.

COMPETING MODELS		*β*	SE	*p*	AICc	ΔAICc	Weighted AICc
Visitors					271.22		1.00
	*Intercept*	−1.297	0.385	<0.001			
	*Visitors*	0.035	0.001	<0.0001			
Null model					330.38	59.2	0.00

Comparison of the two competing models built to study the relationship between the number of large carnivore attacks on humans and recreation visitors over time in national parks in the US as a surrogate of the visitation rates across the entire United States (n = 53). Summary of fitted parameters is shown for the most parsimonious candidate model. Competitive models are ranked from the lowest AICc value (best model) to the highest one. Year was not considered in this analysis because it was highly correlated with the number of visitors (Spearman rank correlation r_s_ = 0.926, P < 0.001). Response variable: Number of attacks per year–Negative binomial distribution error. Deviance = 0.692.

