# Supplementary material for: Human behaviour can trigger large carnivore attacks in developed countries
Source: Sci Rep. 2016 Feb 3;6:20552. doi: 10.1038/srep20552 (PMC4738333; doi:10.1038/srep20552)
Supplement: Supplementary Information [file srep20552-s1.pdf]

Human behaviour can trigger large carnivore attacks in developed countries

Vincenzo Penteriani[1,2], María del Mar Delgado[3], Francesco Pinchera[4], Javier Naves[1], Alberto Fernández-Gil[1], Ilpo Kojola[5], Sauli Härkönen[6], Harri Norberg[6], Jens Frank[7], José María Fedriani[1,8], Veronica Sahlén[9], Ole-Gunnar Støen[9], Jon E. Swenson[9,10], Petter Wabakken[11], Mario Pellegrini[4], Stephen Herrero[12] & José Vicente López-Bao[2,7]

[1] Department of Conservation Biology, Estación Biológica de Doñana, C.S.I.C., c/Américo Vespucio s/n, 41092 Seville, Spain

[2] Research Unit of Biodiversity (UMIB, UO-CSIC-PA), Oviedo University - Campus Mieres, 33600 Mieres, Spain

[3] Metapopulation Research Centre, University of Helsinki, FI-00014 Helsinki, Finland

[4] C.I.S.D.A.M., Via S. Liberata 1, Rosello (CH) I-66040, Italy

[5] Natural Resources Institute Finland, P.O. Box 16, FI-96301 Rovaniemi, Finland

[6] Finnish Wildlife Agency, Sompiontie 1, FI-00730 Helsinki, Finland

[7] Grimsö Wildlife Research Station, Department of Ecology, Swedish University of Agricultural Sciences, 73091 Riddarhyttan, Sweden

[8] Centre for Applied Ecology "Prof. Baeta Neves", Institute Superior of Agronomy, University of Lisbon, Tapada da Ajuda, 1349-017 Lisboa, Portugal

[9] Department of Ecology and Natural Resource Management, Norwegian University of Life Sciences, Postbox 5003, NO-1432 Ås, Norway

[10] Norwegian Institute for Nature Research, PO Box 5685 Sluppen, NO-7485 Trondheim, Norway

[11] Faculty of Applied Ecology and Agricultural Sciences, Hedmark University College, Evenstad, NO-2480, Koppang, Norway

[12] Faculty of Environmental Design, University of Calgary, Calgary, Alberta, Canada T2T 2Y2

Extended Data 1. Some examples of the way human attacks are presented and described by media

The Guardian. (2008) (Date of access: 24/09/2015)

http://www.theguardian.com/world/2008/jul/24/russia.wildlife;

Genesio, J., Natural Unseen Hazards Blog . (2014) (Date of access: 24/09/2015)

https://naturalunseenhazards.wordpress.com/2014/09/20/;

Lallanilla, M., Live Science. (2014) (Date of access: 24/09/2015)

http://www.livescience.com/43339-tigers-animals-attack-eat-kill-human-prey.html;

Sky News. (2013) (Date of access: 24/09/2015)

http://news.sky.com/story/1102544/mans-remains-found-in-killed-bears-stomach;

Wolf Facts. (2014) (Date of access: 24/09/2015)

http://wolffacts.org/do-wolves-attack-humans.html;

Gillman, O., Daily Mail, Mail online. (2014) (Date of access: 24/09/2015)

http://www.dailymail.co.uk/news/article-2723818/Wolf-pack-attacks-Chinese-villagers-tearing-victim-s-ear-leaving-two-seriously-injured.html)

Extended Data Figure

Extended Data Figure 1 | Temporal trends in the age of victims in North America. The age of victims increased significantly (Extended Data Table 2) in the last few decades for those species that were involved in most of the predatory attacks (black bears, cougars and coyotes). (The black pictures have been downloaded from 123RF ROYALTY FREE STOCK PHOTOS (http://www.123rf.com), Images: ID 9068368, Pavel Konovalov; ID 5248826, Vukasin Ilic; ID 6240519, Sergey Yakovlev; ID 6500877, Mila Gligoric).

Extended Data Figure 2A | Trends in the number of attacks by party size. In the last few decades, a slight but insignificant (Extended Data Table 4) increase has been recorded in the number of attacks on humans when in a group for most large carnivore species (dark grey = victim alone; light grey = young in a group; black = adult in a group). Parties: (1) victim alone; (2) young in a group: the victim was a young person (<16 years old) in a group of adults (2 or more people); and (3) adult in a group: the victim was an adult (>16 years old) in a group of adults (2 or more people). (The black pictures have been downloaded from 123RF ROYALTY FREE STOCK PHOTOS (http://www.123rf.com), Images: ID 9068368, Pavel Konovalov; ID 5248826, Vukasin Ilic; ID 6240519, Sergey Yakovlev; ID 6500877, Mila Gligoric).

Extended Data Figure 2B | Size of the human party during an attack (all species pooled). We observed a slight but insignificant (Extended Data Table 3) temporal increase in attacks on both young people (<16 years old) in a group of adults (2 or more people) and adults (>16 years old) in a group of adults.

Extended Data Figure 3 | Trends in large carnivore harvest across the US and Canada. Although the bounty system of persecution has been prohibited since the 1960s-1970s, large carnivore populations continue to be harvested in most of the areas were attacks on humans have been recorded. In fact, in some areas harvest has increased over time, e.g. cougars in Alberta and Colorado, brown bears in Alaska and black bears in Alaska and British Columbia. Control kills denote cougars and brown bears killed by Conservation Officers or anyone else as an official response to large carnivore-human conflicts or interactions.

Extended Data Figure 4A | Increasing trends of different aspects of human leisure activities in natural areas. During the last few decades, an increase in specific human

activities related to the outdoors in American natural areas can be observed: participants in wildlife-related recreation (wildlife watching, fishing and hunting), number of anglers and hunters, days spent angling and fishing (combined), people participating in bicycling, camping and snow skiing, as well as people engaging in outdoor activities (note that all of these activities are related to situations where large carnivore attacks occurred). The U.S. Fish and Wildlife Service has conducted these national surveys at approximately 5-year intervals since 1955, resulting in a 46-year record of the number of anglers and hunters in a given year. The number of wildlife-watching participants can be traced over 21 years, as wildlife watching became part of the survey in 1980. Trends show that the number of anglers increased at nearly twice the rate of the U.S. population growth from 1955 to 2001. The U.S. population increased by 71% while the fishing population increased by 130% during that period. The number of hunters also increased over the 46-year period, but not at a rate equal to population growth. The number of hunters increased 31% from 1955 to 2001.

Extended Data Figure 4B| Increasing trends of different aspects of human leisure activities in natural areas in the US. Millions of dollars spent by Americans on sporting goods related to activities in natural areas during the last two decades. The increasing trends in almost all the leisure activities related to the presence of humans in natural areas is also reflected by the increasing amount of money spent on goods related to some of the activities associated with situations in which large carnivore attacks occurred, e.g. hiking, mountain biking and skiing.

Extended Data Figure 4C | Increasing trends of the different aspects of human leisure activities in natural areas. Trends of human activity in natural areas of Sweden.

Extended Data Figure 1

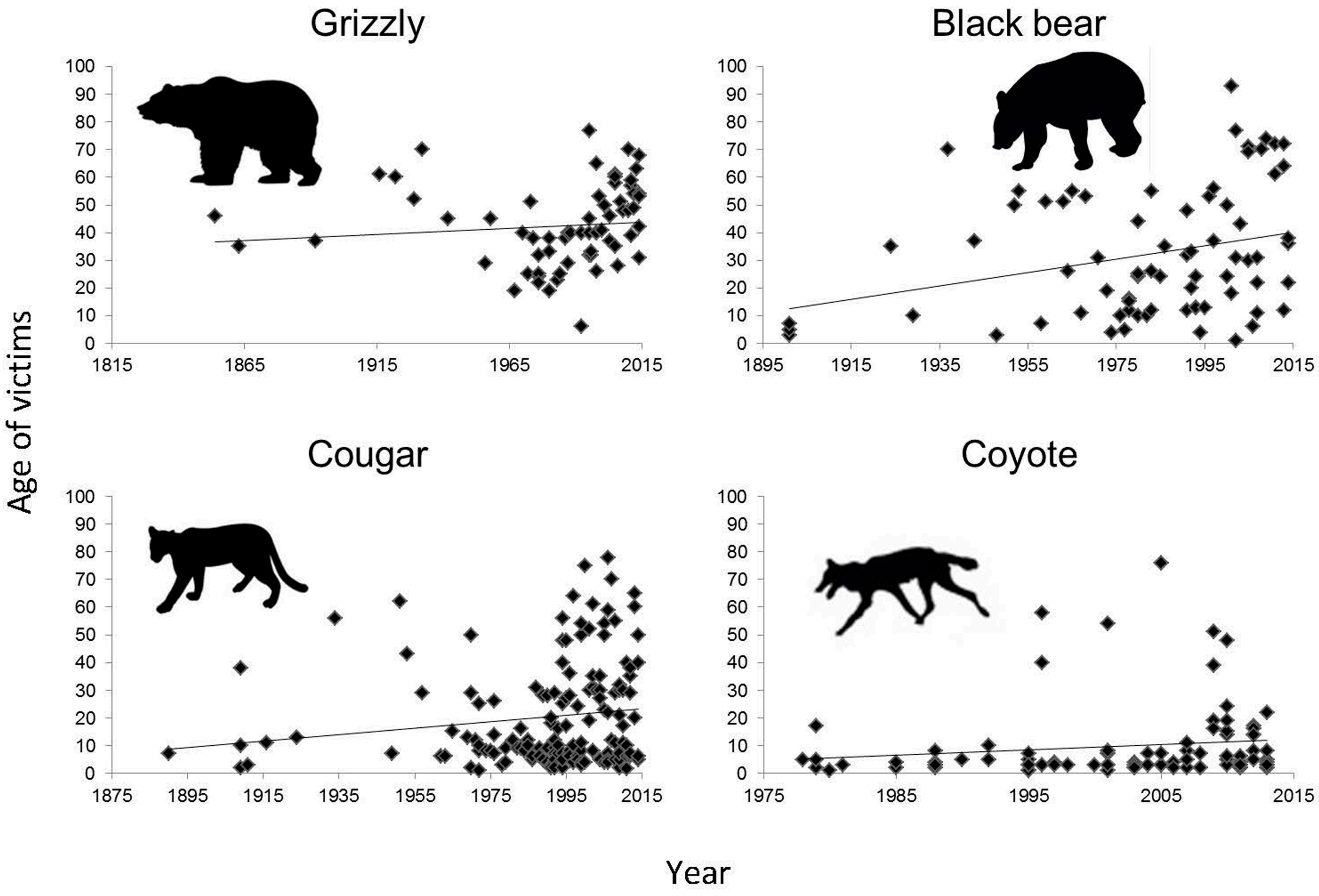

Extended Data Figure 2A

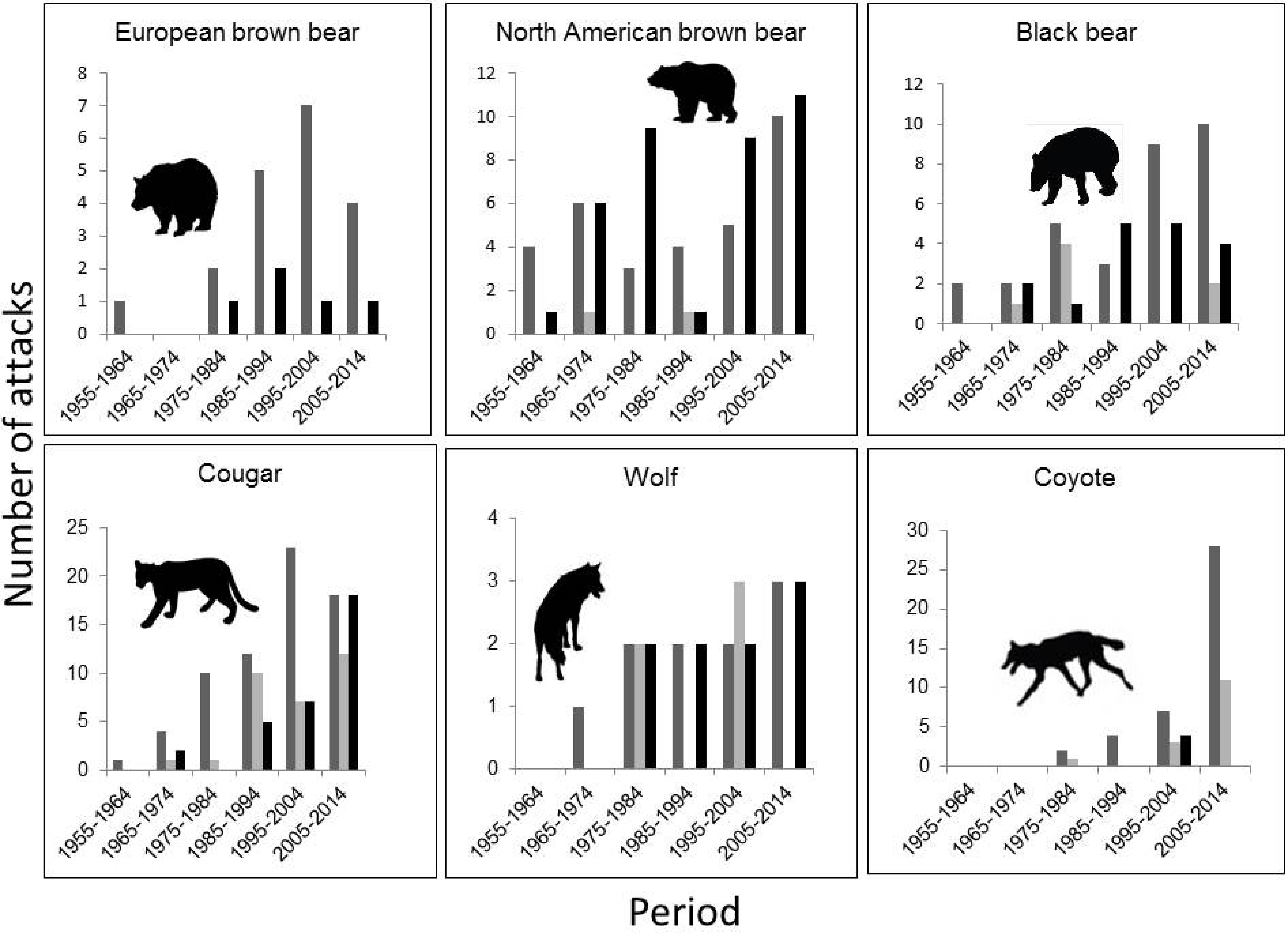

Extended Data Figure 2B

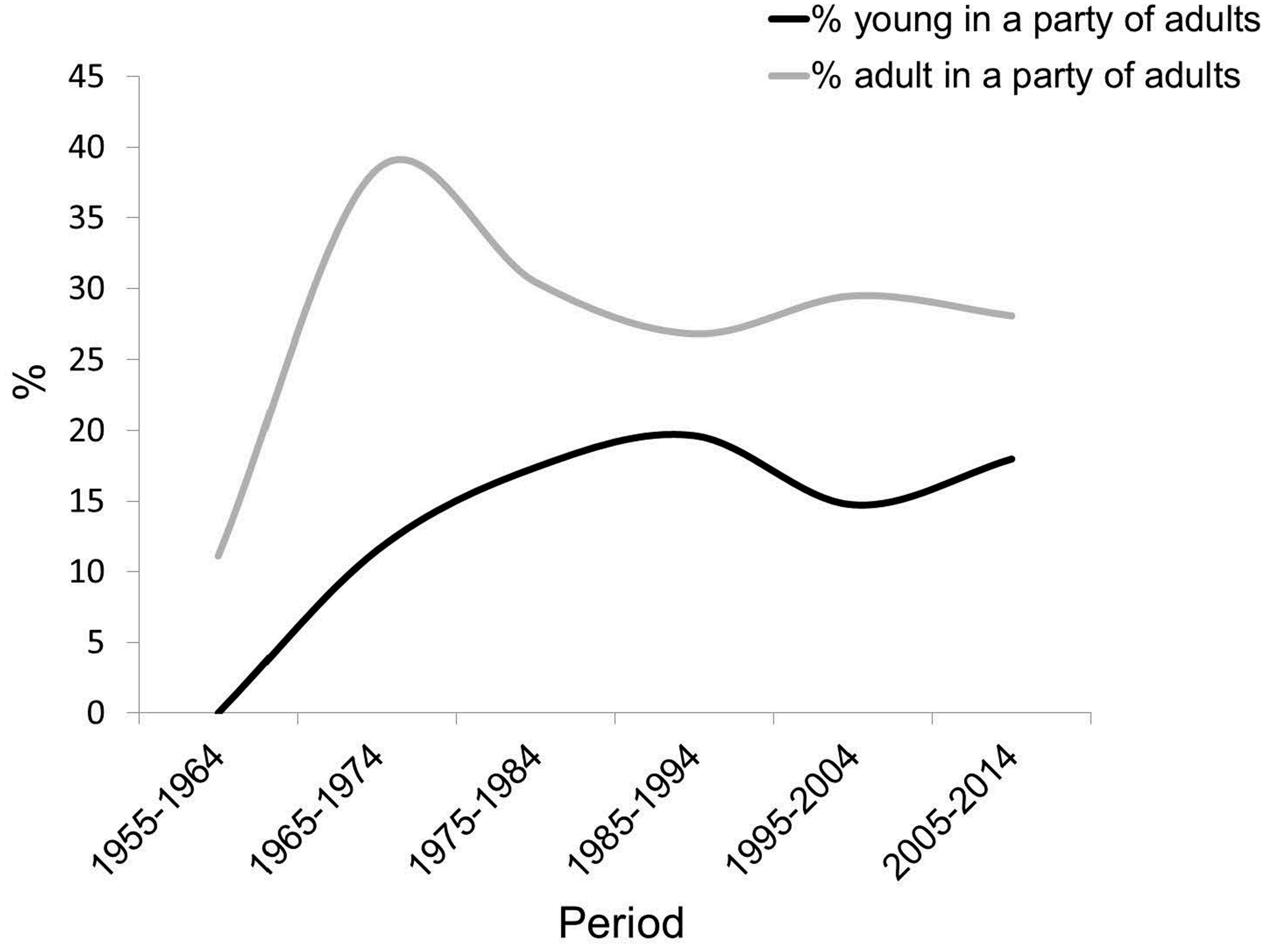

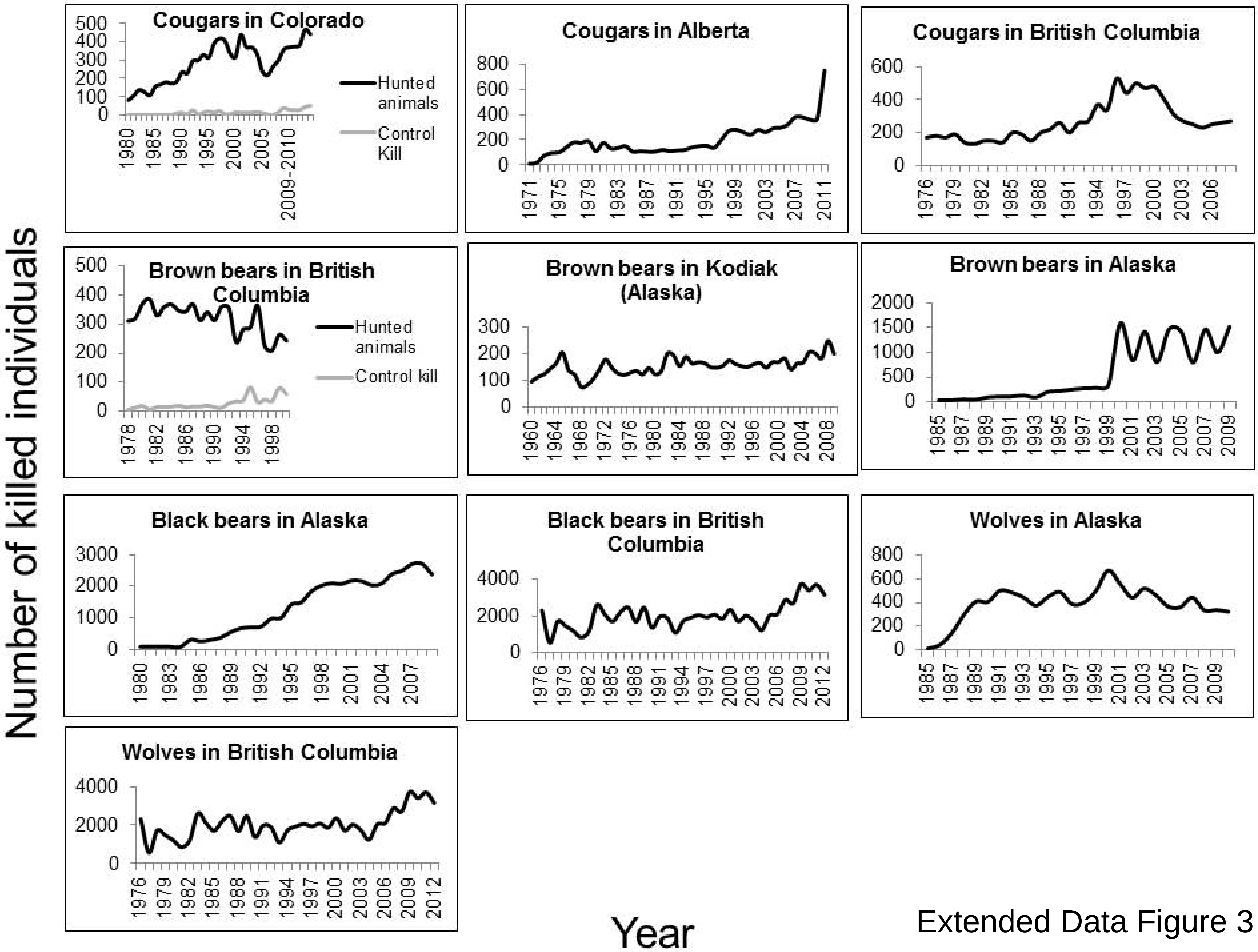

Extended Data Figure 3

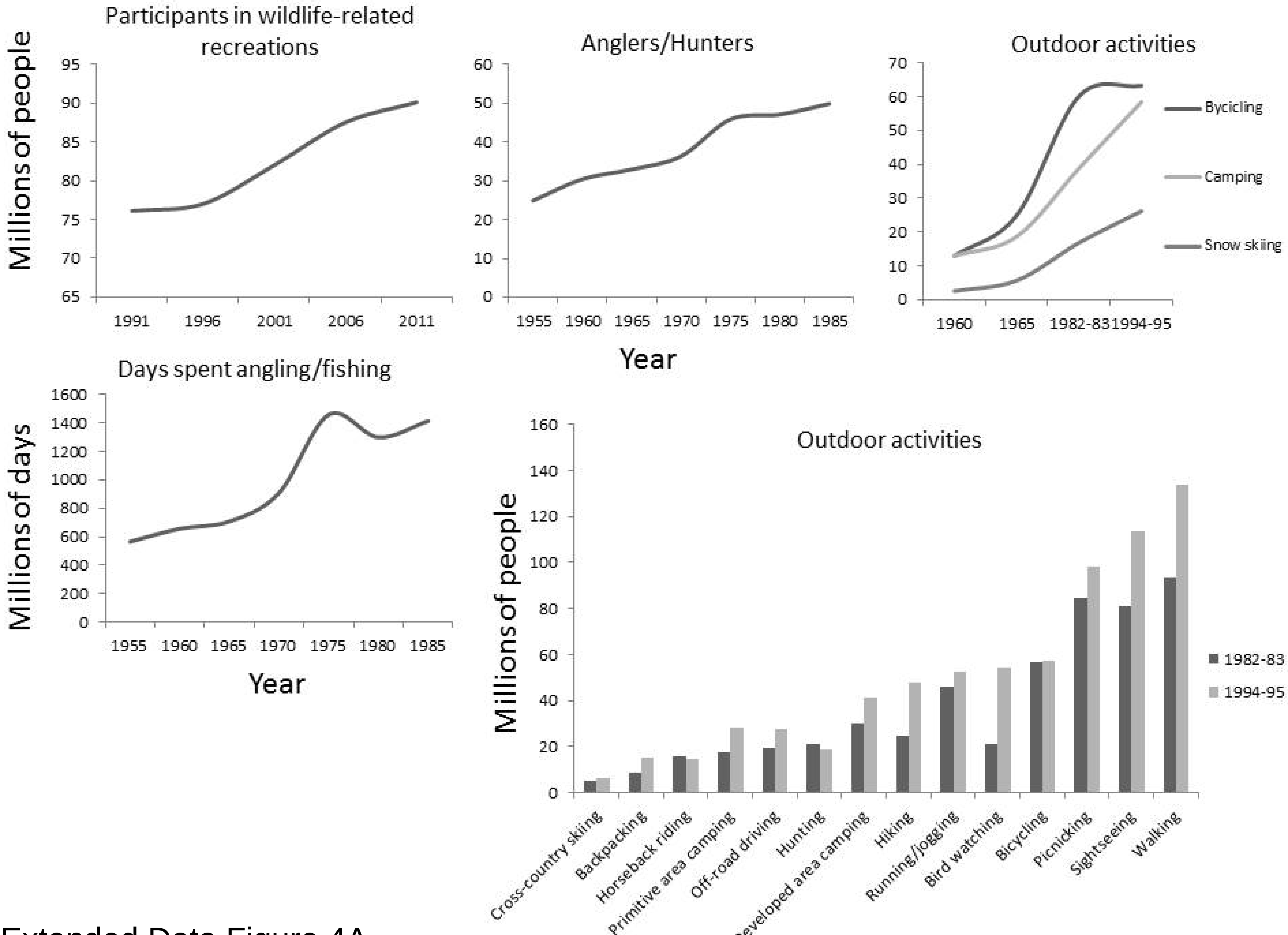

Extended Data Figure 4A

Extended Data Figure 4B

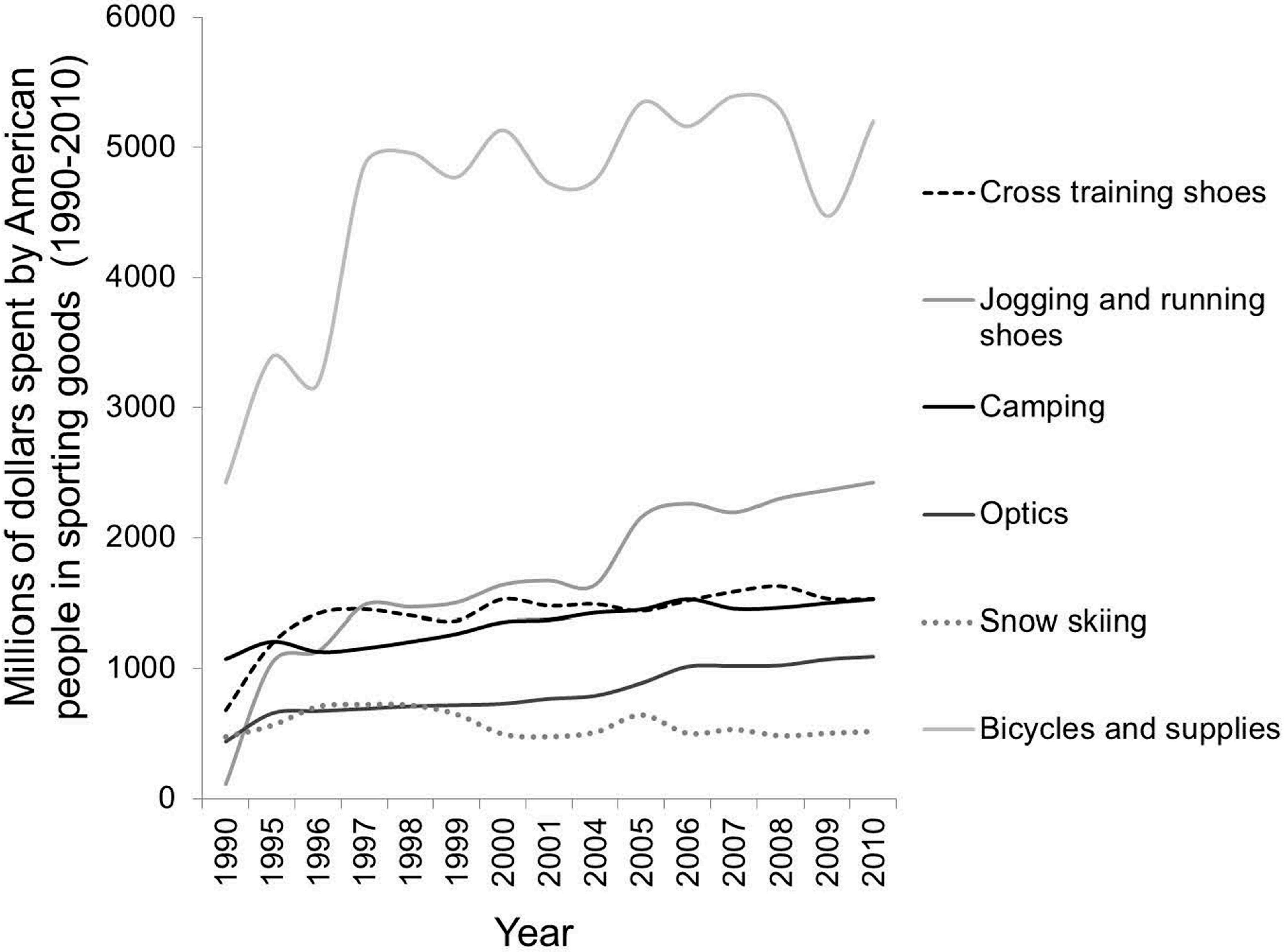

Extended Data Figure 4C

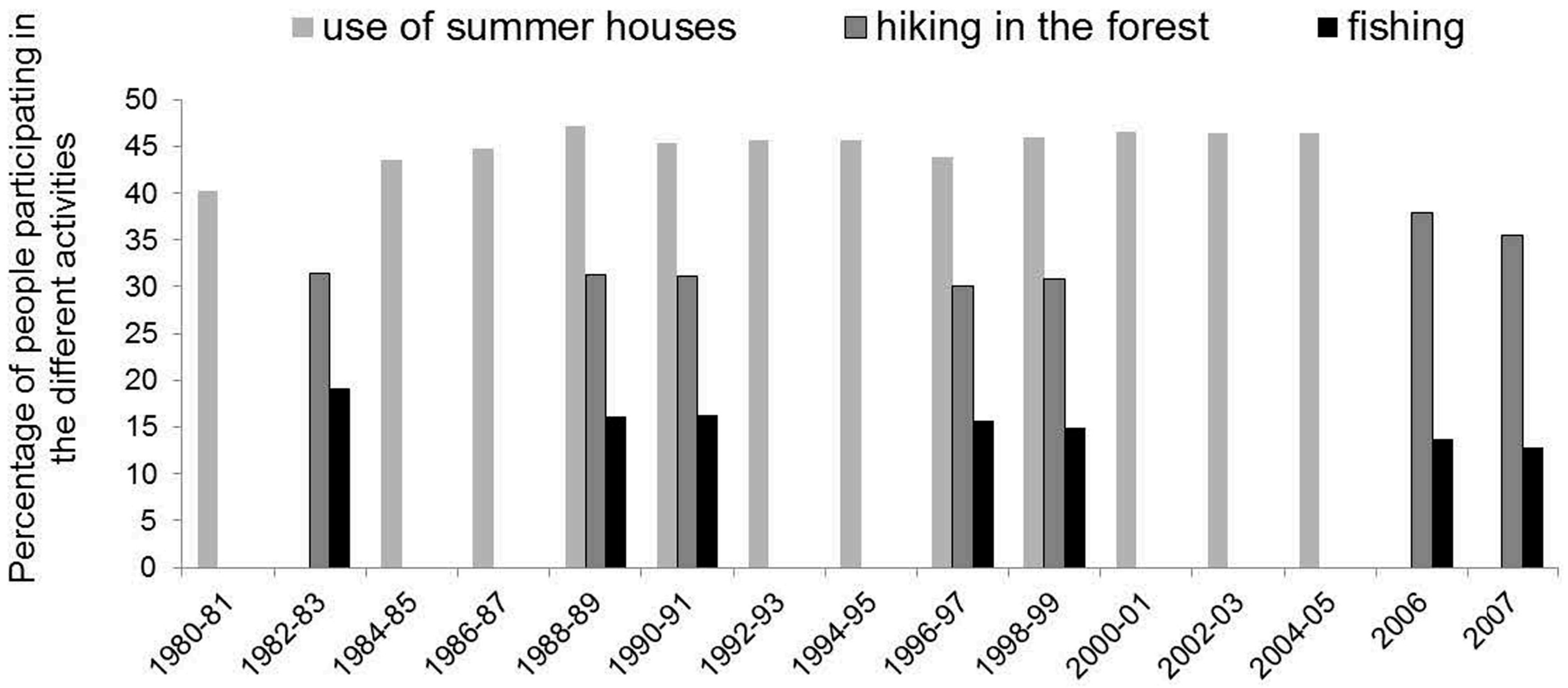

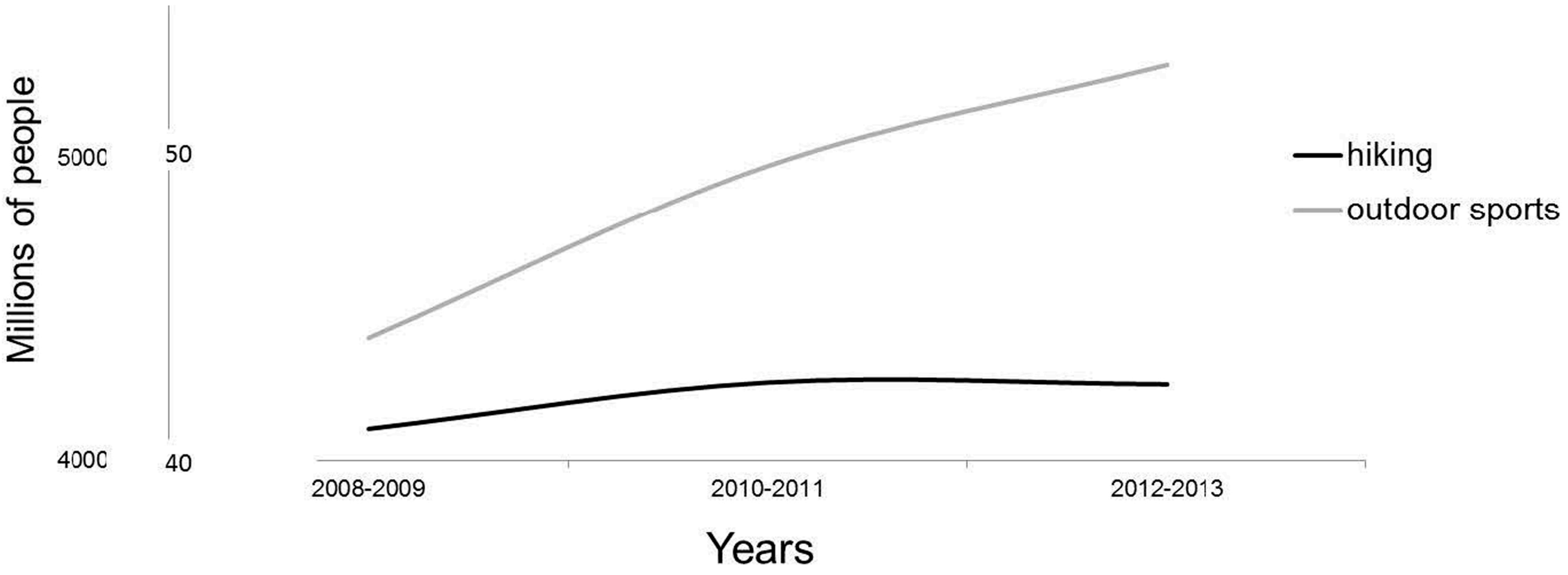

Extended Data Table 1 | Variation in the number of large carnivore attacks on humans over time and among species. Comparison of the five competing models built to study the relationship between large carnivore attacks and time and species (n = 231). Summary of fitted parameters is shown for the most parsimonious candidate model. Competitive models are ranked from the lowest AICc value (best model) to the highest one.

**COMPETING MODELS**		***β***	**SE**	***p***	**AICc**	**ΔAIC**	**Weighted AIC**
Year + Species + Year: Species					888.59		0.95
	Intercept	-13.878	14.580	0.341			
	Year	0.007	0.007	0.318			
	Species	-10.508	3.721	0.005			
	Year: species	0.005	0.002	0.004			
Year + Species					894.3	5.73	0.05
Year					913.4	24.81	0.00
Species					954.9	66.28	0.00
Null model					976.1	87.49	0.00

Explanatory variable: Number of attacks per year – Negative binomial distribution error
Deviance = 0.370

Extended Data Table 2 | Variation of the age of victims in large carnivore attacks in relation to time and species. Comparison of the five competing models built to study the variation in the age of victims over time and across species (n = 188). Summary of fitted parameters is shown for the most parsimonious candidate model. Competitive models are ranked from the lowest AICc value (best model) to the highest one.

**COMPETING MODELS**		***β***	**SE**	***p***	**AICc**	**ΔAIC**	**Weighted AIC**
Year + Species					482.6		0.55
	Intercept	-28.769	9.613	3.14E-03			
	Year	0.016	0.005	7.59E-04			
	Species	-0.497	0.046	<2e-16			
Year + Species + Year: Species					483.1	0.44	0.44
Species					492.1	9.52	0.00
Year					572.7	90.12	0.00
Null model					573.2	90.57	0.00

Explanatory variable: Log (age of victims) – normal distribution error
Adjusted R-squared = 0.389

Extended Data Table 3 | Variation of the party size targeted in an attack over time and across species. Comparison of the five competing models built to study the variation of the party size targeted in an attack over time and across species (n = 371). Summary of fitted parameters is shown for the most parsimonious candidate model. Competitive models are ranked from the lowest AICc value (best model) to the highest one. Party size was classified into three categories: Party 1) the victim was alone; Party 2) the victim was a young person (< 16 years old) in a group of adults (2 or more people); and Party 3) the victim was an adult (>16 years old) in a group of adults (2 or more people).

**COMPETING MODELS**		***β***	**SE**	**AICc**	**ΔAIC**	**Weighted AIC**
Species				711.6		0.67
	Intercept (party 2)	-2,564	0,467			
	Intercept (party 3)	0,238	0,312			
	Species (party 2)	0,333	0,103			
	Species (party 3)	-0,254	0,085			
Year + Species + Year: Species				714.2	2.60	0.18
Year + Species				714.6	2.94	0.15
Null model				735.1	23.52	0.00
Year				738.3	26.64	0.00

Explanatory variable: Party size (3 levels) – multinomial distribution error
Deviance = 0.038

Extended Data Table 4 | Relationship between large carnivore attacks and the number of recreation visitors in national parks in the US over time. Comparison of the five competing models built to study the relationship between large carnivore attacks and outdoor activities over time (n = 53). Summary of fitted parameters is shown for the most parsimonious candidate model. Competitive models are ranked from the lowest AICc value (best model) to the highest one.

**COMPETING MODELS**		**β**	**SE**	***p***	**AICc**	**ΔAIC**	**Weighted AIC**
Visitors					261.92		1.00
	Intercept	0.826	0.094	<0.0001			
	Visitors	-0,007	0.001	<0.0001			
Null model					326.1	326.1	0.00

Explanatory variable: Number of recreation visitors in the US per year – Gamma distribution error
Deviance = 0.684
